# Enhanced BSA Detection Precision: Leveraging High-Performance Dual-Gate Ion-Sensitive Field-Effect-Transistor Scheme and Surface-Treated Sensing Membranes

**DOI:** 10.3390/bios14030141

**Published:** 2024-03-13

**Authors:** Yeong-Ung Kim, Won-Ju Cho

**Affiliations:** Department of Electronic Materials Engineering, Kwangwoon University, Gwangun-ro 20, Nowon-gu, Seoul 01897, Republic of Korea; jobs0506@kw.ac.kr

**Keywords:** bovine serum albumin (BSA), biosensor, ion-sensitive field-effect transistor (ISFET), dual-gate (DG) structure, capacitive coupling effect, SnO_2_ sensing membrane, surface treatment

## Abstract

Bovine serum albumin (BSA) is commonly incorporated in vaccines to improve stability. However, owing to potential allergic reactions in humans, the World Health Organization (WHO) mandates strict adherence to a BSA limit (≤50 ng/vaccine). BSA detection with conventional techniques is time-consuming and requires specialized equipment. Efficient alternatives such as the ion-sensitive field-effect transistor (ISFET), despite rapid detection, affordability, and portability, do not detect BSA at low concentrations because of inherent sensitivity limitations. This study proposes a silicon-on-insulator (SOI) substrate-based dual-gate (DG) ISFET platform to overcome these limitations. The capacitive coupling DG structure significantly enhances sensitivity without requiring external circuits, owing to its inherent amplification effect. The extended-gate (EG) structure separates the transducer unit for electrical signal processing from the sensing unit for biological detection, preventing chemical damage to the transducer, accommodating a variety of biological analytes, and affording easy replaceability. Vapor-phase surface treatment with (3-Aminopropyl) triethoxysilane (APTES) and the incorporation of a SnO_2_ sensing membrane ensure high BSA detection efficiency and sensitivity (144.19 mV/log [BSA]). This DG-FET-based biosensor possesses a simple structure and detects BSA at low concentrations rapidly. Envisioned as an effective on-site diagnostic tool for various analytes including BSA, this platform addresses prior limitations in biosensing and shows promise for practical applications.

## 1. Introduction

The ongoing global pandemic caused by COVID-19 has created a heightened awareness of biological stability in crucial medical materials, the lack of which is a pressing concern [1,2,3,4,5]. This awareness is particularly pronounced with regard to vaccines and has led to an increased demand for the evaluation of biological stability using precise detection equipment capable of assessing various biological factors [6,7,8,9]. Bovine serum albumin (BSA) is a protein that is widely used in biochemistry and molecular biology and is a vital raw material for vaccine production. However, its potential allergenicity in humans necessitates the strict limitation of BSA content. The World Health Organization (WHO)’s recommended BSA limit is ≤50 ng per vaccine [10,11,12,13]. Consequently, biosensors that are capable of precisely detecting BSA levels are critically needed.

Traditional methods that have been used for BSA detection, such as the Bradford protein assay, have drawbacks such as extended detection times and the requirement for specialized equipment [14,15,16]. To address these challenges, the ion-sensitive field-effect transistor (ISFET) has emerged as an effective solution that offers rapid detection, affordability, and portability [17,18,19,20,21]. Developed by Bergveld in the early 1970s, the ISFET is a type of biosensor that analyzes the electrical characteristics of the detection material based on the interaction between the sensing membrane and ions. Its compatibility with CMOS processes enables its mass production and miniaturization. However, ISFETs have critical limitations owing to the Nernst limit (59.14 mV/pH at room temperature), which results in reduced sensitivity [22,23,24,25].

To address these challenges, we propose an alternative dual-gate field-effect transistor (DG-FET)-based BSA-detection biosensor platform. To enhance its detection capabilities, we utilized a dual-gate (DG) structure and capacitive coupling effects [26,27,28,29,30,31]. The capacitive coupling effect is determined by the capacitance ratio between the top-gate (TG) oxide and the bottom-gate (BG) oxide and achieves high sensitivity without requiring additional circuits, owing to its inherent amplification effect. Additionally, we adopted an extended-gate (EG) structure in which the transducer unit for electrical signal processing and the sensing unit for biological detection are separate. This offers advantages such as the prevention of chemical damage to the transducer and the accommodation of a variety of biological analytes [32,33,34,35]. The economic viability and ease of EG production make it readily replaceable; thus, it is an excellent means of detecting irreversible reactions [36,37,38,39]. Furthermore, its simple structure provides significant advantages for rapid and diverse transformation to detect biochemical signals via various surface treatments. We performed a vapor-phase surface treatment with (3-Aminopropyl) triethoxysilane (APTES) to incorporate BSA detection capability [40,41,42]. In addition to the commonly used SiO_2_ sensing membrane, the EG sensing membrane incorporated a SnO_2_ sensing membrane, which has been reported by other biochemical sensor studies to display high sensitivity and facilitate comparative analysis. By leveraging the high detection efficiency of the SnO_2_ sensing membrane, we aimed to achieve precise detection capabilities and extend its application to BSA detection [43,44,45,46].

In this study, we meticulously evaluated characteristics such as the sensing capability and long-term stability of the developed DG-FET-based BSA-detection biosensor platform [47,48,49] with the aim to validate its suitability for detecting various biological molecules and compounds to ensure safe manufacturing and quality assessment of pharmaceuticals. Based on the results, we envision that the proposed sensor platform will play a pivotal role in enhancing patient safety, public health protection, and the overall advancement of biosensing technologies.

## 2. Materials and Methods

### 2.1. Material Specifications

The materials used in this study and their specifications are as follows: glass substrate (Corning 7059; Corning Inc., Corning, NY, USA), bovine serum albumin (BSA; purity = 98%, Sigma-Aldrich, St. Louis, MO, USA), triethylamine (C_6_H_15_N; purity = 98%, Sigma-Aldrich, St. Louis, MO, USA), sodium chloride (NaCl; purity = 99%, Sigma-Aldrich, St. Louis, MO, USA), calcium chloride (CaCl_2_; purity = 99%, Sigma-Aldrich, St. Louis, MO, USA), and potassium chloride (KCl; purity = 99%, Sigma-Aldrich, St. Louis, MO, USA).

### 2.2. Fabrication of the DG-FET as Transducer for the BSA-Detection Biosensor Platform

In this study, a double-gate FET, which acts as a pivotal transducer unit in the BSA-detection biosensor platform, was fabricated on a silicon-on-insulator (SOI) substrate. To optimize the amplification potential of capacitive coupling, tantalum oxide (Ta_2_O_5_), which is a high-*k* material, was selected as the TG dielectric. To minimize the interface defects with the silicon channel, Ta_2_O_5_ was deposited on the silicon dioxide (SiO_2_) layer. The SOI substrate comprises a 100 nm thick p-type top Si layer and a 200 nm thick buried oxide (BOX) layer. The Radio Corporation of America (RCA) cleaning process was used to eliminate contaminants on the substrate. Subsequently, a 150 nm thick dummy oxide layer was deposited using radiofrequency (RF) magnetron sputtering. The source and drain regions were fabricated on the dummy oxide layer using photolithography and wet-etching techniques employing a 30:1 buffered oxide etchant (BOE) solution as the etchant. Phosphosilicate glass (PSG) doping was performed using a thermal diffusion process on the patterned source and drain regions.

**Figure 1:**
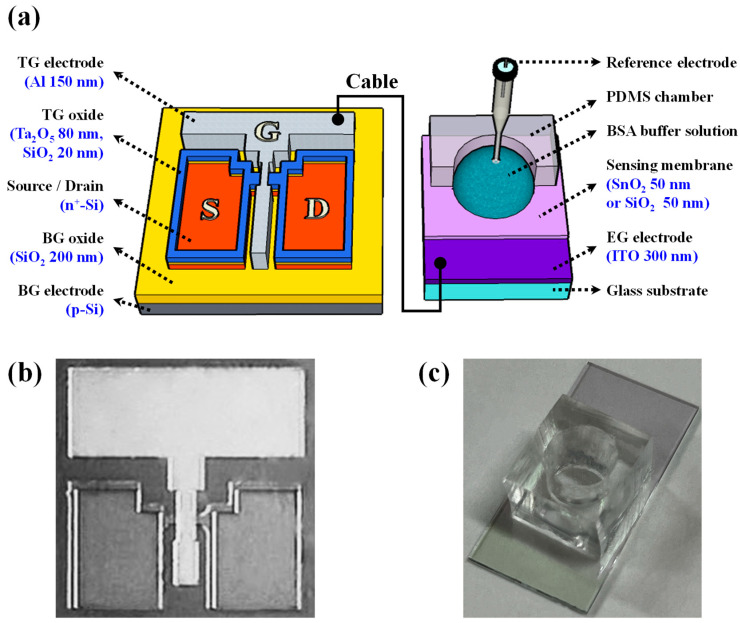
(**a**) Schematic representation of the overall structure of the dual-gate field-effect transistor (DG-FET)-based bovine serum albumin (BSA)-detection biosensor platform. (**b**) Optical microscopy image of the fabricated DG-FET unit. (**c**) Photograph of the BSA-detection extended-gate (EG) unit.

The PSG solution was applied via spin-coating at a speed of 2000 rpm for a duration of 20 s, and then solidified on a hot plate at 200 °C for 10 min. To activate the dopant, rapid thermal annealing (RTA) was conducted at 950 °C for a duration of 30 s in an O_2_/N_2_ gas environment. Following activation, the remaining PSG and dummy oxide layers were eliminated utilizing a 30:1 BOE. Subsequently, the active area was delineated through reactive ion etching (RIE) after photolithography patterning, resulting in a channel width of 10 μm. A high-*k* engineered dielectric layer comprising hybrid SiO_2_ and Ta_2_O_5_ layers was applied to the TG oxide. To improve the interface properties with the Si channel and reduce the leakage current of Ta_2_O_5_, a 20 nm thick SiO_2_ layer was first deposited, followed by an 80 nm thick Ta_2_O_5_ layer. To achieve a 5-fold amplification ratio, the hybrid layer used as the TG oxide was designed to have an equivalent oxide thickness (EOT) of less than 1/5 compared with the SiO_2_ layer used as the BG oxide. Subsequently, a TG electrode was fabricated using the deposition of a 150 nm thick Al thin-film layer using an electron-beam evaporator. To enhance the electrical properties of this electrode, forming gas annealing (FGA) was carried out at 450 °C for 30 min (in a 5% H_2_/N_2_ gas ambient). Figure 1a illustrates the overall structure of the DG-FET-based BSA-detection biosensor platform, and Figure 1b shows the optical microscopy image of the fabricated DG-FET, providing visual insight into the sophisticated structure of the proposed platform. Figure 1c shows a photograph of the BSA-detection EG, providing visual insight into the implementation of the DG-FET-based biosensor platform. These detailed fabrication steps ensured the creation of a highly sensitive and stable transducer for this BSA-detection biosensor platform.

### 2.3. DG Structure and Capacitive Coupling Effect of Transducer

**Figure 2:**
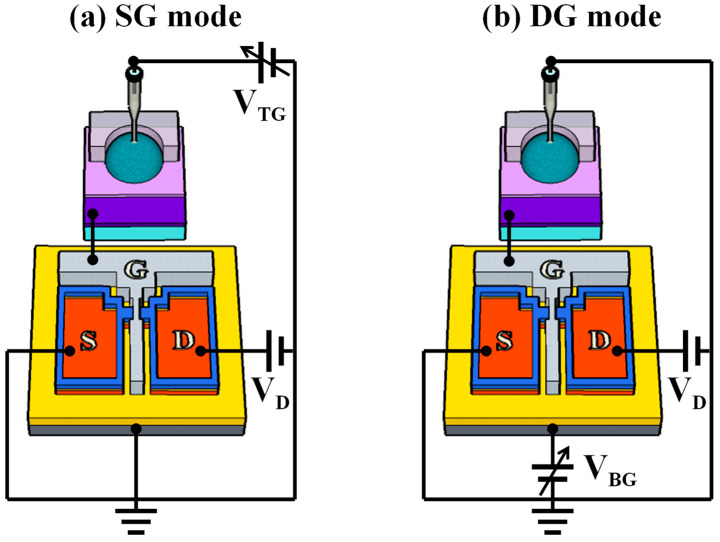
Simplified equivalent circuits of a DG-FET-based sensor platform in (**a**) single-gate (SG) and (**b**) dual-gate (DG) modes of operation.

Figure 2 illustrates the simplified equivalent circuit of the DG-FET-based sensor platform in single-gate (SG) and DG operation modes. As this interpretation is based on the operational principle of the DG-FET without incorporating a surface-treated EG, we elucidated this as in the case of a basic ISFET. In an ISFET featuring a DG structure, the sensing process is segregated into two operation modes: the SG mode and the DG mode. In SG mode, only one gate is utilized, whereas in DG mode, both gates are employed simultaneously. During SG mode operation, surface potential detection and voltage sweeping activities are conducted exclusively at the TG, while the BG remains grounded. In this configuration, the threshold voltage shift (Δ*V_TH_*) is determined solely based on the change in surface potential (Δ*ψ*), as expressed in Equation (1) [50,51,52]:

(1) $$\Delta V_{TH}^{TG}=-\Delta \psi$$

Conversely, in the DG mode, voltage sweeping is performed using the bottom gate, whereas changes in the surface potential are detected using the top gate. In this case, a capacitive coupling effect arises between the dielectric layers of the TG and BG. The BG electrode amplifies the alteration in surface potential generated by the TG electrode, surpassing the Nernst limit. Here, the movement in the transfer curve due to Δ*ψ* is amplified according to the capacitance ratio of the TG and BG dielectric layers. The relationship between $V_{TH}^{TG}$ and $V_{TH}^{BG}$ is expressed in terms of the V_TH_ of the TG and BG, as shown in Equation (2). By rearranging Equations (1) and (2), we obtain Equation (3) [53,54,55]:

(2) $$\Delta V_{TH}^{BG}=\frac{C_{TOX}}{C_{BOX}}\cdot \Delta V_{TH}^{TG}$$

(3) $$\Delta V_{TH}^{BG}=-\frac{C_{TOX}}{C_{BOX}}\cdot \Delta \psi$$

Therefore, the change in surface potential induced during detection is amplified at the bottom gate by the ratio of $C_{TOX}/C_{BOX}$.

### 2.4. Fabrication of the EG for the BSA-Detection Biosensor Platform

**Figure 3:**
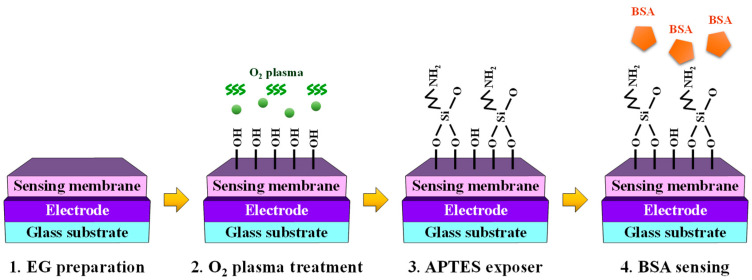
Schematic representation of the process of fabricating the BSA-detection extended-gate (EG) structure for the biosensor platform.

A glass substrate (dimensions: 1.5 cm × 2.5 cm) was used to fabricate the EG sensing unit. The electrode for the EG was deposited using a 300 nm thick indium tin oxide (ITO) layer, and two types of EG units were prepared by adopting SiO_2_ or SnO_2_ as the sensing membrane. The thickness of both sensing membranes was set to 50 nm, and they were deposited using the same RF magnetron sputtering method. After physical assembly, the sensing membranes were surface functionalized for BSA detection. Initially, the fabricated EG was subjected to a 30 s O_2_ plasma treatment to induce the formation of OH groups on the surface [56,57]. Subsequently, APTES exposure facilitated amino functionalization. This specific functionalization was aimed at enabling the detection of BSA (a polymer protein) and was achieved by exposing APTES to the OH groups developed on the surface of the sensing membrane through the vapor-phase reaction method [40,41]. To minimize the impact of humidity, a 5 L desiccator was purged with argon gas before the commencement of the process. Two trays were placed inside the desiccator, and 30 μL of APTES and 10 μL of triethylamine were pipetted onto each tray. After preparation, the O_2_ plasma-treated EGs were placed in a desiccator, and APTES and triethylamine were removed after 120 min of incubation. The desiccator was filled with argon gas. Finally, the EGs were stored in a desiccator for 48 h to cure the APTES coatings [58,59]. The manufacturing process of BSA-detectable EG is illustrated in Figure 3.

### 2.5. Device Characterization

The electrical properties of the proposed sensor platform were assessed during sensing operations employing an Agilent 4156 B precision semiconductor parameter analyzer (Agilent Technologies, Santa Clara, CA, USA). The sensing unit and the transducer unit were connected using an RG58A 9222 electrical cable (BELDEN, St. Louis, MO, USA). To mitigate potential interference from external factors such as light and noise, all measurements were performed within a light-attenuating enclosure. The reference electrode (Horiba 2080-06T; Kyoto, Japan) was submerged in the BSA buffer solution within the PDMS reservoir located on the EG unit and linked to the TG of the DG-FET. The sensitivity of the DG-FET-based BSA-detection biosensor platform was determined by measuring the change in the transfer curve at a drain current of 1 nA (constant-current method) corresponding to the BSA concentration of the buffer solution. This measurement method involves designating the point where a specific current value is reached during the detection of the analyte as the ‘reference voltage (V_REF_)’ and tracking its changes. For sensitivity measurements, BSA buffer solutions with concentrations ranging from 10 nM to 100 μM were prepared by dissolving BSA powder in deionized water. Sensitivity extraction was defined as the difference in V_REF_ when detecting deionized water (zero BSA concentration) and each concentration of the BSA buffer solution; this was used to quantify the response of the sensor to varying BSA concentrations. Additionally, the non-ideal effects of the proposed sensor platform were assessed by evaluating the drift effects. To evaluate reliability during continuous measurements, drift effects were investigated by exposing the sensing membrane of the EG to a 100 μM BSA buffer solution for a duration of 10 h and monitoring the ΔV_REF_ between the initial and final values.

## 3. Results and Discussion

### 3.1. Electrical Characteristics of the DG-FET

**Figure 4:**
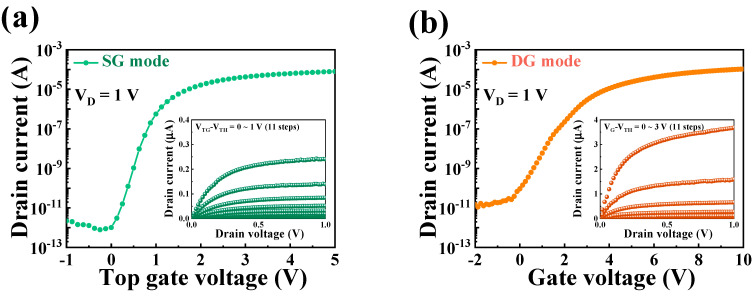
Electrical characteristics of the DG-FET in (**a**) SG and (**b**) DG operation modes.

Figure 4 illustrates the electrical characteristics of the proposed transducer device in both SG and DG operation modes. The electrical characteristics were evaluated using transfer (I_D_–V_G_) and output (I_D_–V_D_) curve measurements. For the transfer curve measurements, V_D_ was set to 1 V in both SG and DG modes. In the output curve measurements, V_G_–V_TH_ was varied from 0 to 1 V in 11 steps (1 step = 0.1), and the V_D_ sweep range was set from 0 to 1 V. Table 1 summarizes the electrical parameters of the fabricated transducer based on data from Figure 4. In the SG mode, the device exhibited a subthreshold swing (SS) of 137.76, an ON/OFF current ratio (I_ON_/I_OFF_) of 1.52 × 10^8^, and a field-effect mobility (μ_FE_) of 140.51. In contrast, in the DG mode, it demonstrated an SS of 213.56, an I_ON_/I_OFF_ ratio of 8.44 × 10^7^, and a μ_FE_ of 384.55, respectively. In the SG mode, in which operation occurs through the TG, the characteristics of the TG oxide with high-*k* materials play a significant role. This confirms the excellent SS characteristics stemming from the outstanding electric field transferability of the TG oxide layer. In the DG mode, enhanced channel control was achieved while operating through both TG and BG, which allows for a higher current value.

### 3.2. Sensing Characteristics of the DG-FET-Based BSA-Detection Biosensor Platform

**Figure 5:**
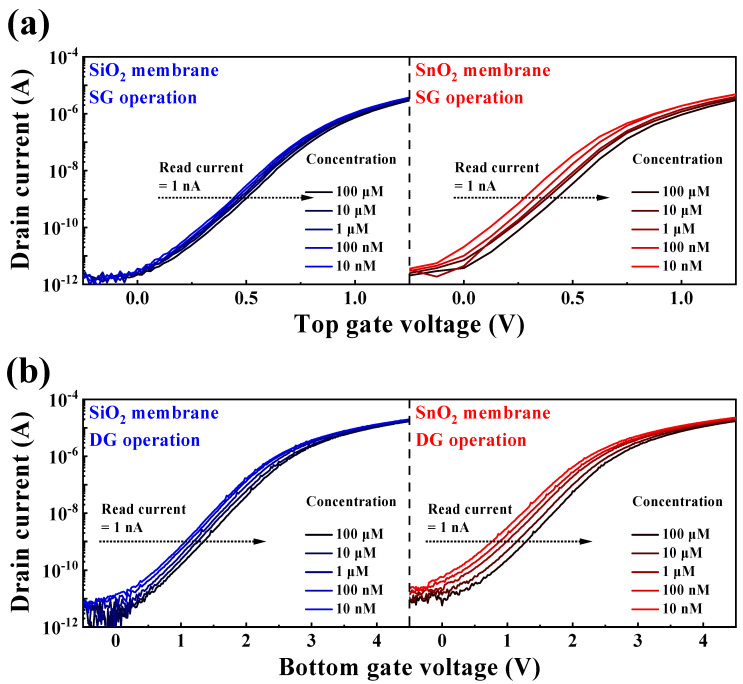
Transfer characteristic curves as a function of BSA concentration for (**a**) SG and (**b**) DG sensing modes.

The stringent WHO guidelines, which stipulate a maximum allowable BSA concentration of ≤50 ng/vaccine, pose a considerable challenge for electrochemical detection in solutions at extremely low BSA concentrations [10]. However, the engineered DG-FET-based BSA-detection biosensor platform is distinguished by its capacity to precisely self-amplify minute potentials, which effectively demonstrates its proficiency in detecting BSA levels with remarkable sensitivity. An EG with integrated BSA sensing capabilities was used to perform the measurements, as outlined in the illustration of the surface treatment process in Figure 3. The BSA detection characteristics of the prepared biosensor platform were evaluated with respect to BSA concentration, and the ΔV_REF_ for various BSA buffer solutions was extracted at 1 nA, as shown in Figure 5. At concentrations below 10^−9^ M, the linearity of the transfer curve shift was not maintained, making accurate detection impossible. Therefore, the detection limit was set to 10^−8^ M, and concentrations were extracted in the range of 10^−8^ to 10^−4^ M. Figure 5a,b show the transfer characteristic curves as a function of BSA concentration for the SG and DG sensing modes, respectively. Notably, for both the SiO_2_ and SnO_2_ sensing membranes, the observed ΔV_REF_ in the DG mode surpasses that in the SG mode. This observation underscores that the BSA detection capability in DG mode sensing, especially with the SnO_2_ sensing membrane used in this study, outperforms SG mode sensing using the conventional SiO_2_ sensing membrane, owing to the amplification effect of capacitive coupling in the DG mode.

**Figure 6:**
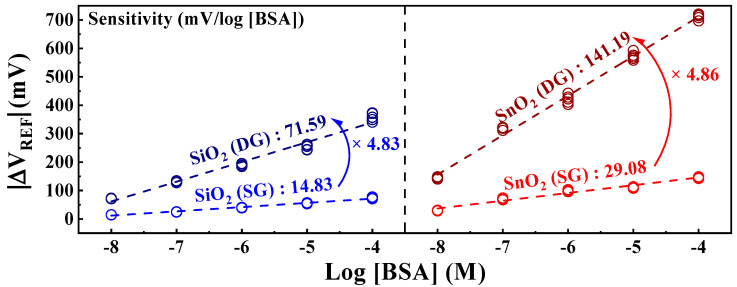
Sensitivity of the DG-FET-based BSA-detection biosensor platform as a function of BSA concentration.

The BSA sensitivity extracted from Figure 5 is shown in Figure 6. The sensitivity values were determined as the averages of the measurement results from five samples. Owing to the irreversible nature of the detection response in the BSA-detection EG, each individual EG was used as a disposable unit for precise detection. The SiO_2_ sensing membrane exhibited sensitivities of 14.83 mV/log [BSA] in SG mode and 71.59 mV/log [BSA] in DG mode. Meanwhile, the SnO_2_ sensing membrane demonstrated sensitivities of 29.08 mV/log [BSA] in SG mode and an impressive 141.19 mV/log [BSA] in DG mode. Both sensing membranes demonstrated approximately 4.8 times increased sensitivity owing to the amplification effect of the capacitive coupling in the DG mode. Notably, the SnO_2_ sensing membrane, with exceptional detection capabilities, achieved a remarkable sensitivity of 141.19 mV/log [BSA] after amplification. These results indicate that the proposed DG-FET-based biosensor effectively detects BSA, proving its efficiency as a biosensor platform with high sensitivity for detecting minute quantities of biomolecules via self-amplification.

### 3.3. Selectivity Characteristics of the DG-FET-Based BSA-Detection Biosensor

**Figure 7:**
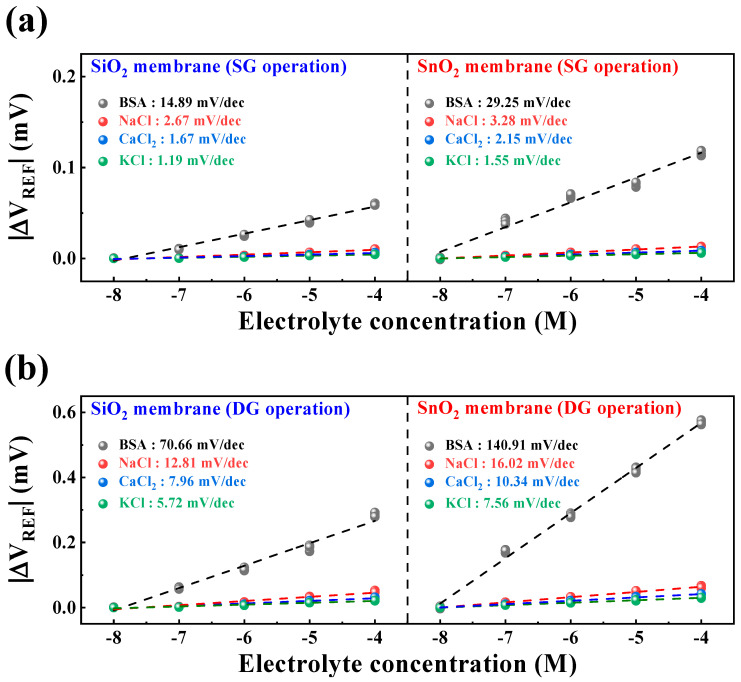
Selectivity characteristics of the DG-FET-based BSA-detection biosensor platform for (**a**) SG and (**b**) DG sensing modes.

To assess the selective detection capability of the fabricated DG-FET-based BSA-detection biosensor for biological analytes, sensitivity characteristics were compared for NaCl, CaCl_2_, KCl, and BSA buffer solutions. Figure 7a,b illustrate the sensitivity characteristics in the SG and DG sensing modes for each buffer solution. The sensitivity values were calculated as the averages of the measurement results from five samples. The maximum interference ratios of other ions to BSA were 17.93% in SG mode and 18.13% in DG mode for SiO_2_ samples, and 11.21% in SG mode and 11.37% in DG mode for SnO_2_ samples. This reaffirms that the SnO_2_ sensing membrane can provide stable and accurate BSA detection capability compared with the SiO_2_ sensing membrane. Moreover, the maximum interference ratios of other ions to BSA were sufficiently small to be negligible, indicating the potential utility of the proposed biosensor platform capable of BSA detection. The numerical values extracted from Figure 7 for the selectivity characteristics are presented in Table 2.

### 3.4. Reliability and Stability of the DG-FET-Based BSA-Detection Biosensor

**Figure 8:**
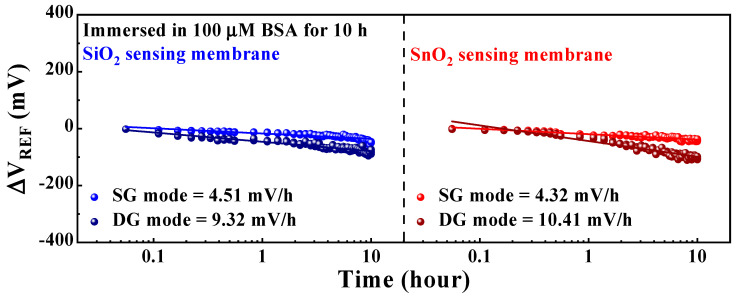
Drift effects of the proposed DG-FET-based BSA-detection biosensor platform.

This section discusses the reliability and stability of the DG-FET-based BSA-detection biosensor platform, focusing on the non-ideal effects and drift rates observed during prolonged sensing operations. Ensuring the capability to detect BSA at levels recommended by the WHO demands not only high sensitivity but also the stability of the sensor platform itself, which facilitates consistent sensing operations. Therefore, non-ideal effects were evaluated through repetitive and sustained operations under various environmental conditions. Figure 8 illustrates the drift effect of the proposed DG-FET-based BSA-detection biosensor platform. The parameters were measured for 10 h after applying a 100 μM BSA buffer solution, and the ΔV_REF_ of the initial and final points were compared. The drift effect values were determined as the averages of the measurements from five samples. The drift effects in SG and DG modes for the SiO_2_ sensing membrane were 4.51 mV/h and 9.32 mV/h, and those for the SnO_2_ sensing membrane were 4.32 mV/h and 10.41 mV/h, respectively. In both cases, the drift effects were lower in the SG mode than in the DG mode.

Table 3 presents a comprehensive summary of the observed non-ideal effects and sensitivity values under various measurement conditions. The standard deviation (σ) values for the extracted data are indicated below the sensitivity and drift effect values. To facilitate a quantitative comparison, the sensitivity-to-drift effect ratio for each condition is also provided.

**Figure 9:**
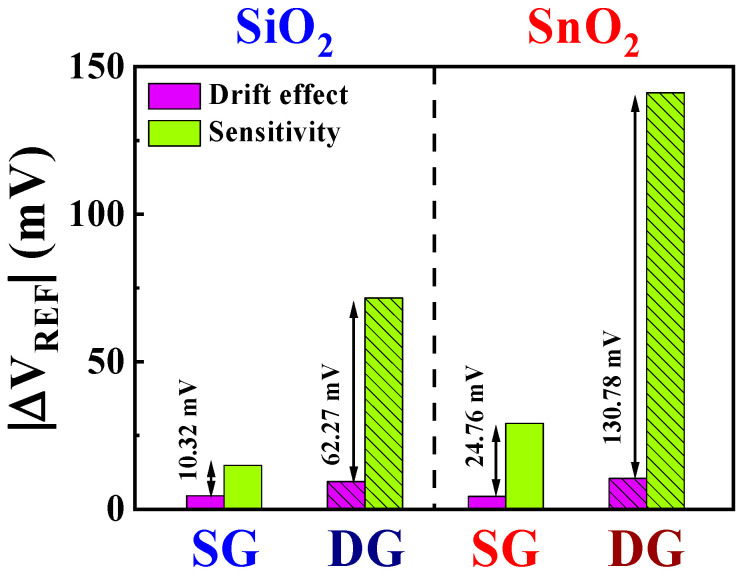
Numerical comparison of the drift effect and sensitivity of the DG-FET-based BSA-detection biosensor platform.

Figure 9 presents the numerical comparative data of the non-ideal effects and sensitivity values based on Table 2. Compared with that of the SiO_2_ sensing membrane, the numerical value of the drift effect for the SnO_2_ sensing membrane is higher. Furthermore, the drift effect in the DG mode surpasses that in the SG mode. However, considering the significantly higher sensitivity gain achieved through the amplification effect, this difference is negligible. Using SnO_2_ sensing membranes and leveraging amplification minimizes the impact of non-ideal effects, leading to the establishment of a stable sensor platform. This highlights the exceptional detection capabilities at low BSA concentration and the high stability of the proposed DG-FET-based BSA-detection biosensor platform.

## 4. Conclusions

In this study, we introduce a novel DG-FET-based biosensor platform for the precise detection of BSA. The proposed platform overcomes the limitations of traditional ISFETs by incorporating a DG structure into an SOI substrate. The capacitive coupling effect between the TG and BG significantly enhances sensitivity without requiring additional external circuits. This innovative design achieves rapid and sensitive detection of low concentrations of BSA, satisfying the critical requirements mandated by the WHO for applications in vaccine production. The DG-FET biosensor demonstrates excellent sensitivity, with a remarkable amplification effect in the presence of SnO_2_ sensing membranes. This platform exhibits superior sensitivity and stability, outperforming traditional ISFETs. Compared with sensors produced using conventional methods, which exhibit a sensitivity of 14.83 mV/log [BSA], this sensing platform achieves a significantly higher sensitivity (approximately 9.52 times) of 141.19 mV/log [BSA]. Sensitivity-to-drift effect ratio analysis revealed the robustness of the proposed biosensor, particularly when the SnO_2_ sensing membranes were used in the DG mode. Our findings suggest that the developed DG-FET-based biosensor can potentially detect various biological analytes with high precision. Hence, future studies are required to extend the capacity of the proposed biosensor to detect various biological molecules and compounds to ensure the continued improvement in the stability and reliability of biosensing technology. We believe that the significant advances presented in this study could potentially revolutionize biosensor applications in terms of biological stability and detection and contribute to the ongoing efforts to enhance the accuracy and efficiency of biosensors, which in turn would improve safety in pharmaceutical manufacturing and quality assessment, especially in the context of global health challenges.

## Figures and Tables

**Table 1:** Summary of electrical parameters for the dual-gate field-effect transistor (DG-FET) in single-gate (SG) and dual-gate (DG) operation modes.

Electrical Parameters	V_TH_ (V)	SS (mV/dec)	I_ON_/I_OFF_ (A/A)	μ_FE_ (cm^2^/V·s)
SG mode	0.13	137.76	1.52 × 10^8^	140.51
DG mode	−0.25	213.56	8.44 × 10^7^	384.55

**Table 2:** Summary of selectivity characteristics of the DG-FET-based BSA-detection biosensor platform.

Membrane Type	Operation Mode	Sensitivity [mV/dec]			
		BSA	Na^+^	Ca^2+^	K^+^
SiO_2_	SG mode	14.89 (σ = 0.0021)	2.67 (σ = 0.0012)	1.67 (σ = 0.0032)	1.19 (σ = 0.0044)
	DG mode	70.66 (σ = 0.0119)	12.81 (σ = 0.0037)	7.96 (σ = 0.0021)	5.72 (σ = 0.0016)
SnO_2_	SG mode	29.25 (σ = 0.0061)	3.28 (σ = 0.0018)	2.15 (σ = 0.0011)	1.55 (σ = 0.0019)
	DG mode	140.91 (σ = 0.0121)	16.02 (σ = 0.0046)	10.34 (σ = 0.0028)	7.56 (σ = 0.0037)

**Table 3:** Summary of non-ideal effects and sensitivity values for the DG-FET-based BSA-detection biosensor platform for different sensing modes and sensing membrane types.

Membrane Type	Operation Mode	Sensitivity [mV/dec]	Drift [mV/h]	Sensitivity- Drift Ratio [%]
SiO_2_	SG mode	14.83 (σ = 0.0023)	4.51 (σ = 0.0043)	328.82
	DG mode	71.59 (σ = 0.0137)	9.32 (σ = 0.0047)	768.13
SnO_2_	SG mode	29.08 (σ = 0.0063)	4.32 (σ = 0.0028)	673.15
	DG mode	141.19 (σ = 0.0144)	10.41 (σ = 0.0065)	1356.29

## Data Availability

Data are contained within the article.
